# Purple-leaf tea (*Camellia sinensis* L.) ameliorates high-fat diet induced obesity and metabolic disorder through the modulation of the gut microbiota in mice

**DOI:** 10.1186/s12906-020-03171-4

**Published:** 2020-12-10

**Authors:** Yu-Chun Lin, Hsu-Feng Lu, Jui-Chieh Chen, Hsiu-Chen Huang, Yu-Hsin Chen, Yen-Shuo Su, Chien-Yi Tung, Cheng Huang

**Affiliations:** 1 Department of Biotechnology and Laboratory Science in Medicine, National Yang-Ming University, No. 155, Sec. 2, Linong St., Beitou District, Taipei, 11221 Taiwan; 2 Departments of Clinical Pathology, Cheng Hsin General Hospital, Taipei, 11221 Taiwan; 3 Department of Restaurant, Hotel and Institutional Management, Fu-Jen Catholic University, New Taipei, 24205 Taiwan; 4 Department of Biochemical Science and Technology, National Chiayi University, Chiayi, 60004 Taiwan; 5 Department of Applied Science, National Tsing Hua University, Hsinchu, 30014 Taiwan; 6 Taichung District Agricultural Research and Extension Station, Council of Agriculture, Changhua County, 51544 Taiwan; 7 Tea Research and Extension Station, Council of Agriculture, Taoyuan, 324 Taiwan; 8 Cancer Progression Research Center of National Yang-Ming University, Taipei, 112 Taiwan; 9 Institute of Microbiology and Immunology, National Yang-Ming University, Taipei, 112 Taiwan; 10 Department of Earth and Life Sciences, University of Taipei, Taipei, 11153 Taiwan

**Keywords:** Gut microbiota, Hepatic steatosis, Purple-leaf tea, Insulin resistance, Obesity

## Abstract

**Background:**

Obesity and its associated diseases have become a major world-wide health problem. Purple-leaf Tea *(Camellia sinensis* L.) (PLT), that is rich of anthocyanins, has been shown to have preventive effects on obesity and metabolic disorders. The intestinal microbiota has been shown to contribute to inflammation, obesity, and several metabolic disorders. However, whether PLT consumption could prevent obesity and diet-induced metabolic diseases by modulating the gut microbiota, is not clearly understood.

**Methods:**

In this study, six-week-old male C57BL/6 J mice were fed a normal diet (ND) or a high fat diet (HFD) without or with PLT for 10 weeks.

**Results:**

PLT modulated the gut microbiota in mice and alleviated the symptoms of HFD-induced metabolic disorders, such as insulin resistance, adipocyte hypertrophy, and hepatic steatosis. PLT increased the diversity of the microbiota and the ratio of Firmicutes to Bacteroidetes. f_Barnesiellaceae, g_Barnesiella, f_Ruminococcaceae, and f_Lachnospiraceae were discriminating faecal bacterial communities of the PLT mice that differed from the HFD mice.

**Conclusions:**

These data indicate that PLT altered the microbial contents of the gut and prevented microbial dysbiosis in the host, and consequently is involved in the modulation of susceptibility to insulin resistance, hepatic diseases, and obesity that are linked to an HFD.

## Background

The high prevalence of obesity is a major public health issue in modern societies [1]. Visceral obesity has been shown to be closely associated with chronic inflammation, which may result in insulin resistance, type two diabetes, cardiovascular diseases, and fatty liver diseases [2]. As the most widely consumed beverage after water, tea (*Camellia sinensis*) and its extracts are used as medicinal drinks in Asia and elsewhere with several health promoting effects, especially weight reduction [3–5]. Flavonoids, that are secondary metabolites in many plants, are responsible for the different colors of the leaves and other plant organs. Flavonoids are important plant pigments in flowers, for attracting pollinating animals, and in other plant organs for UV light filtration and symbiotic nitrogen fixation [6]. There are over 5000 flavonoids characterized from various plants, and six groups of flavonoids are classified according to their chemical structures. Among the subgroups of flavonoids, catechins (flava-3-ol) are the most common in foodstuffs and are mainly found in younger tea leaves (12–24% of dry mass). Another subgroup of flavonoids, anthocyanins, which are rarely found in normal green tea leaves, are present in abundance in a purple-leaf tea (PLT) cultivar (*Camellia sinensis* L.).

Purple-leaf Tea (*Camellia sinensis* L., PLT) was once popular as a beverage and food supplement in China and Vietnam. The health benefits of PLT received much more attention in recent years. Previous studies showed that water extraction of PLT has potential antitumor activities through inhibiting human colon tumor cell growth and angiogenesis around tumor [7]. A higher anthocyanin intake has been reported to reduce urinary disorders, dysentery and diarrhea, cerebral problems, and hypertension in human trials [8]. The effect of anthocyanins on lipid metabolism has been approved in different experiments in vitro and in vivo [9]. Anthocyanins and other compounds in PLT might reduce risks of lifestyle related disorders, such as obesity and encephalopathy [9, 10]. However, the effect of PLT on diet-induced obesity and metabolic dysfunction is not well understood.

Food enters the gastrointestinal (GI) tract and interact with the microbiota therein. Recent studies have shown that an imbalance of the intestinal microbiota contributes to inflammation, obesity, and several metabolic disorders [11, 12]. Microbial dysbiosis may compromise the integrity of the colonic barrier and release liposaccharide (LPS) from the surface of Gram-negative bacteria, causing inflammation and disorders. Anthocyanins and other polyphenols are poorly absorbed in the upper GI tract, with high concentrations found in the distal intestine, which and may interact with intestinal microbiota [13, 14]. Anthocyanins are metabolized by some species of bacteria in the gut and have been shown to play a role in the modulation of inflammation by restoring particular species of bacteria and protecting the intestinal barrier [15]. This suggests that the anti-inflammatory effects of phenolic/anthocyanin may involve interaction between the local microbiota and the integrity of the intestine.

In this study, we used the high-fat diet C57BL/6 J mouse model to investigate the effects of PLT on fat-induced adiposity, hepatic steatosis and alteration of gut microbiota. After an HFD, the mice show abnormalities in lipid and glucose metabolism, as well as significant dyslipidemia and markers of hepatic steatosis. We examined whether PLT can ameliorate the diet-induced metabolic disorders and whether the gut microbiota is modulated by the diet and PLT treatment.

## Methods

### Preparation of purple-leaf tea (*Camellia sinensis* L.) (PLT)

PLT *(Camellia sinensis* L. cv. TRES No.25*)* is a new, purple-leaf cultivar and was identified by comparison with the voucher specimen (NRICM-325-CS-01), which is already deposited at the herbarium of the National Research Institute of Chinese Medicine, Taiwan. The seeds of PLT were obtained from the lab of Tea Research and Extension Station, Taiwan, and PLT is grown in Yuchi, Taiwan. One bud and two leaves, which are all purple, were randomly collected from different branches, taken to the processing laboratory, and washed in running water. The PLT leaves were dehydrated and thoroughly homogenized using a homogenizer (D-500, WIGGENS, Germany), ground to a fine powder in liquid nitrogen, packed in polyethylene bags, and stored in a freezer (− 25 °C ± 2 °C).

### Animals

Thirty 5-week-old male C57BL/6 J mice were purchased from BioLASCO Taiwan Co, Ltd. All mice were housed individually under a constant temperature (24 °C) and 12 h light/dark cycle at the Animal Center of the National Yang-Ming University (NYMU), Taipei, Taiwan. Mice fed with a standard diet and adapted to the environment for 1 week were subsequently divided randomly into three groups and fed a normal diet (ND, *n* = 8), high-fat diet (HFD, *n* = 8, 45% fat and 1% cholesterol), or HFD with 1 or 3% (weight for weight) Purple-leaf Tea *(Camellia sinensis* L.) (PLT, *n* = 8) for 10 weeks. At the end of the experimental period, all mice were deeply anesthetized under ketamine and xylazine anesthesia (intramuscular injection of 100 mg/kg body mass and 5 mg/kg body mass, respectively), and sacrificed via cardiac-puncture to collect blood followed by cervical dislocation. The liver and adipose tissue were removed, rinsed with physiological saline, weighed, immediately frozen in liquid nitrogen, and stored until analysis. The use of animals for this research was approved by the Animal Research Committee of the NYMU (IACUC no. 1070213) and all procedures followed the National Institutes of Health guide for the care and use of Laboratory animals (NIH Publications No. 8023, revised 1978) and the guidelines of the Animal Welfare Act, Taiwan.

### Morphology of the liver and fat tissues

The liver and epididymal adipose tissue were removed from each mouse. Samples were subsequently fixed in 10% paraformaldehyde in PBS and embedded in paraffin for hematoxylin and eosin (H&E) staining. Specimens were observed under a light microscope (Carl Zeiss Inc., Germany) at 200× magnification.

### Hepatic-steatosis, and plasma lipids index detection

The total plasma triglyceride (TG), total cholesterol (TC), HDL-cholesterol (HDL-C), glutamic oxaloacetic transaminase (GOT), glutamic pyruvic transaminase (GPT), and Lipase (LIP) levels were detected using enzymatic assay kits and a FUJI DRI-CHEM analyzer (Fujifilm, Tokyo, Japan). The non–HDL-C level was calculated as [(total cholesterol) - (HDL-C) - (TG/5)]. For hepatic-steatosis analysis, a lipid extraction kit from BioVision, USA (Cat. K216–50) was used to extract lipids in liver tissues in accordance with the manufacturer’s protocol. Hepatic triglyceride and cholesterol were determined in the liver tissue by using triglyceride and cholesterol quantitation assay kits from Abcam, UK (Cat. ab65336 and ab65359), respectively, according to the manufacturer’s instruction.

### Blood glucose and intraperitoneal glucose tolerance test (IPGTT)

Every 2 weeks, the 12 h fasting blood glucose concentrations of the mice were measured in tail vein blood using a glucose analyzer (EASYTOUCH, Miaoli County, Taiwan). For the intraperitoneal glucose tolerance test (IPGTT), the analysis was performed during the 10th week after the start of the diet experiments, mice fasted for 12 h were injected intraperitoneally with glucose (1 g/kg body weight), and the blood glucose level was determined in tail vein blood at 0, 30, 60, 90, and 120 min post-glucose injection.

### Gut microbiota analysis

Fecal genomic DNA was extracted using a QIAamp DNA Stool Mini Kit (Qiagen, Germany) according to the manufacturer’s instructions, including a bead-beating step. The metagenome analysis libraries were prepared and sequenced. The V3F/V4R primers (V3F: 5′-CCTACGGGNGGCWGCAG-3′/V4R: 5′-GACTACHVGGGTATCTAATCC-3′) for the hypervariable region of the 16S rRNA gene with overhang sequence were used to generate Illumina 16S library by two-step PCR from genomic DNA. The first stage PCR for amplifying V3-V4 region was performed in duplicate for each DNA sample. The two PCR products of each sample were pooled and subjected to the second PCR using a Nextera XT DNA index kit to add multiplexing indices and Illumina sequencing adapters, according to the 16S metagenomic sequencing library preparation guide. The 16S libraries were pooled and sequenced on a MiSeq with MiSeq V3 reagent paired 300-bp reads. The QIIME 2 software package (version 2018.8) [16] was used to process the raw sequence data. In brief, a total of 3,752,852 sequences were obtained after demultiplexing with q2-demux, with an average sequence length of 300 nt. The mean number of sequences per sample is 150,144 (min: 99,183, max: 196,947). The sequences were replicated, quality filtered and chimera removed with q2-dada2 [17]. Representative sequence sets for each dada2 sequence variant were used for taxonomic classification. Operational taxonomic units (OTUs) were clustered by scikit-learn naive Bayes machine-learning classifier and assigned against the curated Greengenes v13.8 reference database at the QIIME2 website. Microbial diversity was visualized using Principal Coordinate Analysis (PCoA) of Unweighted UniFrac distances. The mean of relative abundance in each group was compared at the phylum, family, and genus levels. Choa1 richness index and Simpson evenness indexes of alpha diversity were calculated by diversity core-metric. Redundancy analysis (RDA) was analyzed by Canoco for Windows 4.5 (Microcomputer Power, NY, USA), which was assessed by MCPP with random permutations.

### Statistical analysis

All values are expressed as the mean ± SD. Area under the curve (AUC) analysis was performed using the trapezoidal method. Student’s *t*-test was used to assess statistical significance. Asterisks indicate that the values were significantly different from the control (*, *P* < 0.05; **, *P* < 0.01, ***, *P* < 0.001.). Microbiome data was analyzed by ANOVA followed by Tukey’s multiple comparison test.

## Results

### HFD-PLT mice have a lower efficiency of food intake, fasting blood glucose and glucose tolerance than HFD mice

**Fig. 1:**
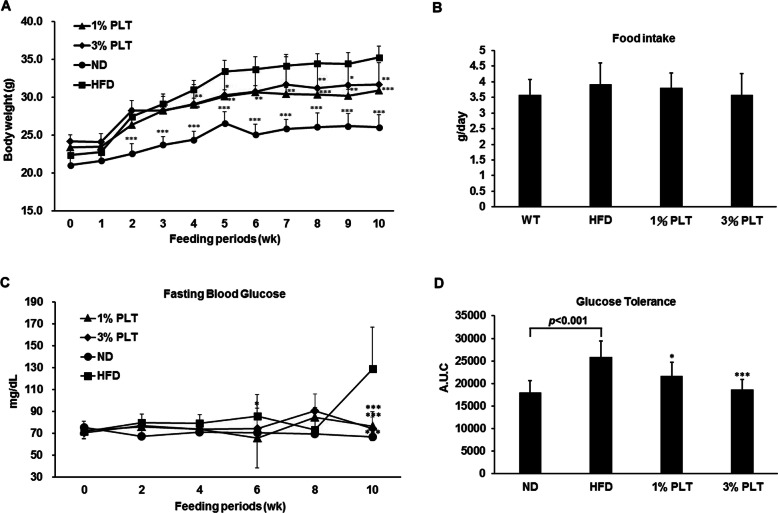
The effect of PLT on body weights, food intake, glucose metabolism and glucose tolerance in C57BL/6 J mice fed an HFD. **a** Changes in body weight. **b** Food intake. **c** Food efficiency ratio (FER). **d** Changes in fasting blood glucose levels after 10 weeks of PLT treatment. **e** Area under the curve (AUC) of IPGTT. Data are shown as means ± SEM. * *p* < 0.05; ** *p* < 0.01; *** *p* < 0 .001 vs. HFD

To determine the effect of PLT on obesity in vivo, an experimental model of HFD-induced obese mice was used. Although the different concentrations of PLT may not show their effects in a dose-dependent manner, the weights of the HFD mice were significantly greater than the ND and HFD-PLT mice after 10 weeks of diet (Fig. 1a). The amount of food consumed by the mice did not differ significantly among the groups (Fig. 1b). Previous studies have confirmed that dietary fat intake is associated with deteriorated insulin function [18]. Therefore, we measured several indices that reflect the incidence of insulin resistance syndrome in mice fed an HFD, with or without PLT supplement. High fasting blood glucose levels in the HFD group are significantly higher than the other groups, which implied an abnormality of insulin function. The levels of high fasting blood glucose were restored in the HFD-PLT groups (Fig. 1c). Moreover, we calculated the area under the curve (AUC) of glucose levels in IPGTT, which is an index of glucose tolerance. The treatment of PLT prevented the impaired glucose tolerance occurred in the HFD group (Fig. 1d). The HFD-induced high fasting blood glucose and glucose tolerance were alleviated when mice were treated with PLT.

### PLT intake decreases fat deposition in the fatty tissues of mice

**Fig. 2:**
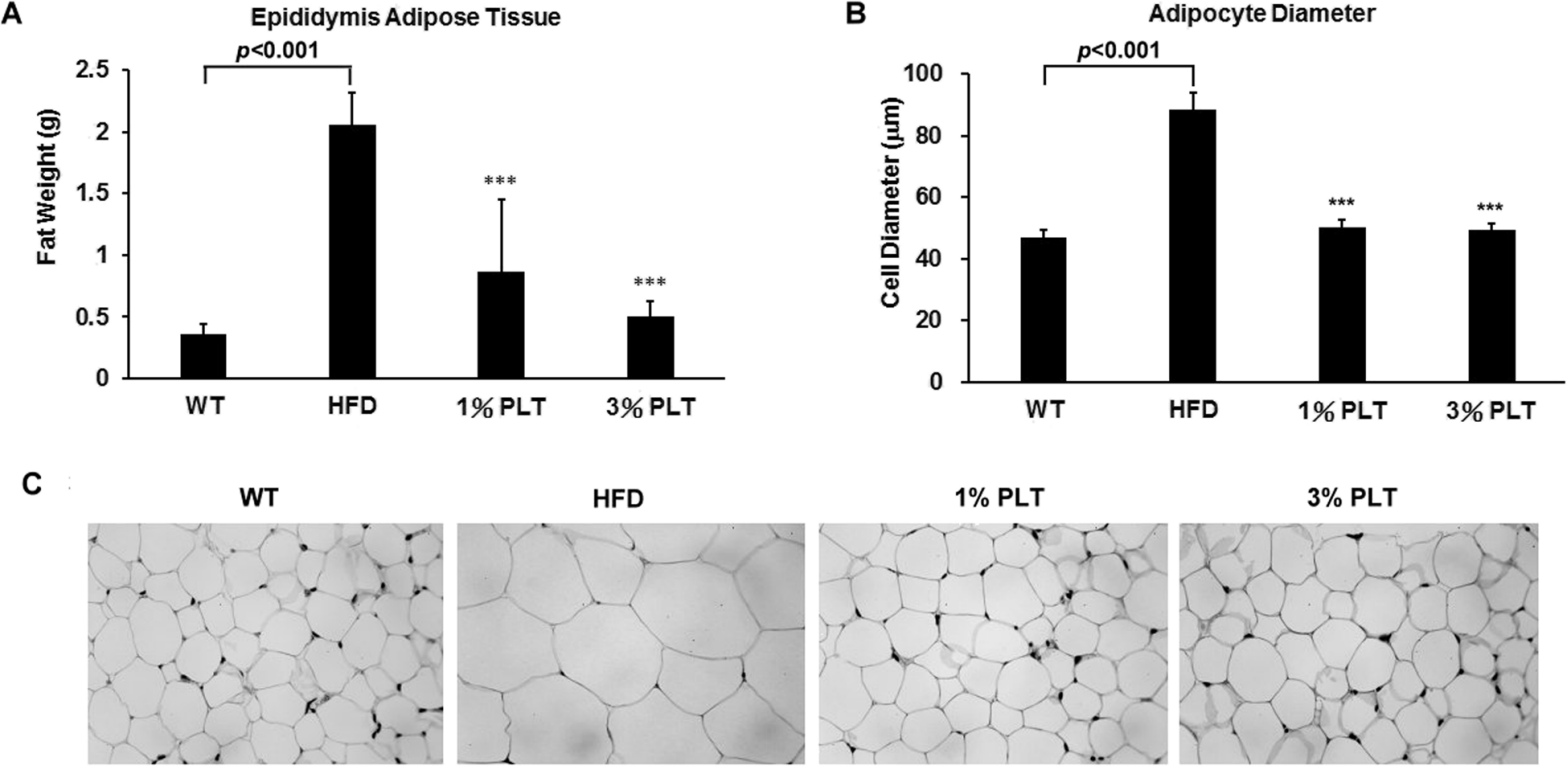
The effect of PLT on fat deposition in C57BL/6 J mice fed an HFD. **a** The weight of Epididymis adipose tissue (EAT). **b** The adipocyte diameters were measured. **c** hematoxylin-eosin staining showed adipocytes in the EAT of mice (original magnification × 200). Data are shown as means ± SEM. * *p* < 0.05; ** *p* < 0.01; *** *p* < 0.001 vs. HFD

Another well-known feature of metabolic syndrome is increase of lipid accumulation in the trunk region, which causes excessive visceral fat deposition [19]. Therefore, we dissected and measured the size of the epididimal adipose tissue (EAT) of mice after 10 weeks of diet. The mass of the EAT in the HFD group was increased to 2.05 ± 0.26 g and significantly greater than the ND (0.36 ± 0.08 g), HFD-1% PLT (0.86 ± 0.58 g) and HFD-3%PLT (0.55 ± 0.12 g) groups (Fig. 2a). In addition, we fixed the EAT in paraffin and stained the tissue with H&E. Tissue specimens were observed under a light microscope and the diameters of the adipose cells were measured. Compared with the adipocyte diameter from the ND group (46.93 ± 2.45 μm), the adipocyte diameter of HFD group was increased to 88.47 ± 5.62 μm, while that of HFD-1% PLT and HFD-3%PLT groups were significantly decreased to 50.17 ± 2.56 μm and 49.35 ± 2.09 μm (Fig. 2b). PLT-fed group had lower cell diameters and smaller cell size (Fig. 2c). This suggests that PLT may affect fat mass in mice.

### PLT administration decreased high fat-induced intracellular lipid accumulation in the livers of the mice

**Fig. 3:**
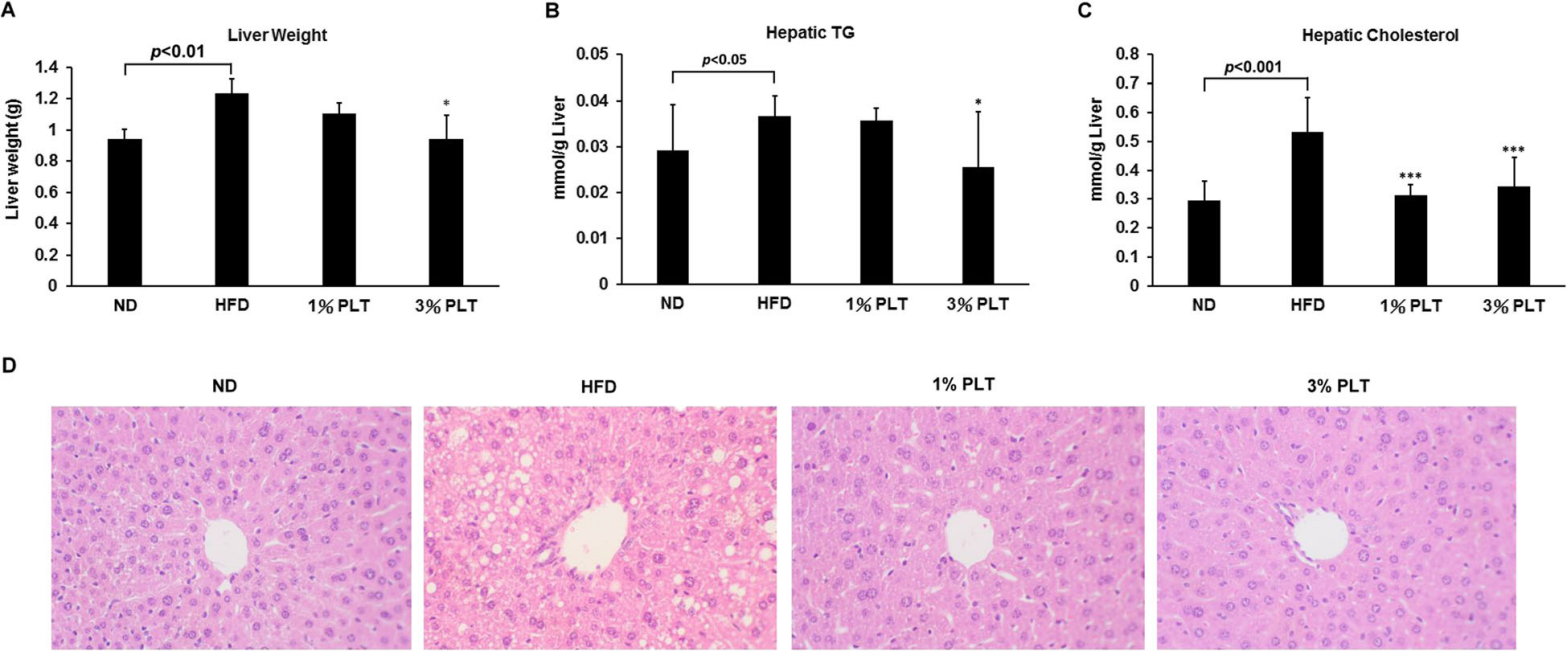
The effect of PLT on lipid accumulation in the livers of C57BL/6 J mice fed an HFD. **a** Liver weight, **b** hepatic TG level, **c** hepatic cholesterol. Data are shown as means ± SEM. * *p* < 0.05; ** *p* < 0.01; *** *p* < 0.001 vs HFD. **d** Hematoxylin and eosin staining of hepatocytes (original magnification × 200)

One of the conditions in metabolic syndrome is non-alcoholic fatty liver disease, which is characterized by triglyceride accumulation in the hepatocytes [20, 21]. First, we weighed the livers and measured the hepatic triglyceride (TG) and hepatic cholesterol levels of the mice to monitor the degrees of lipid deposition in liver. We found that the liver weight of the HFD group (1.23 ± 0.09 g) was significantly higher than the ND (0.94 ± 0.06 g), HFD-1% PLT (1.10 ± 0.07 g) and HFD-3%PLT (0.94 ± 0.15 g) groups (Fig. 3a). The hepatic TG was significantly lower in the HFD-3% PLT group than the HFD group (Fig. 3b). In addition, the hepatic cholesterol levels of the HFD group were higher than the ND and HFD-PLT groups (Fig. 3c). Next, we hypothesized that the increased weight of the livers may be attributable to lipid accumulation. Liver tissue sections of the HFD mice showed accumulation of lipid droplets that displaced the nucleus (macrovesicular steatosis) (Fig. 3d). Lipid accumulation was much lower in the hepatocytes of the PLT-treated groups than the HFD group. The findings implied that the high-fat diet increased lipid levels in the mice and caused lipid accumulation in the livers, and PLT protected the HFD mice from lipid accumulation in the liver.

### Hyperlipidemia was prevented by PLT treatment

**Fig. 4:**
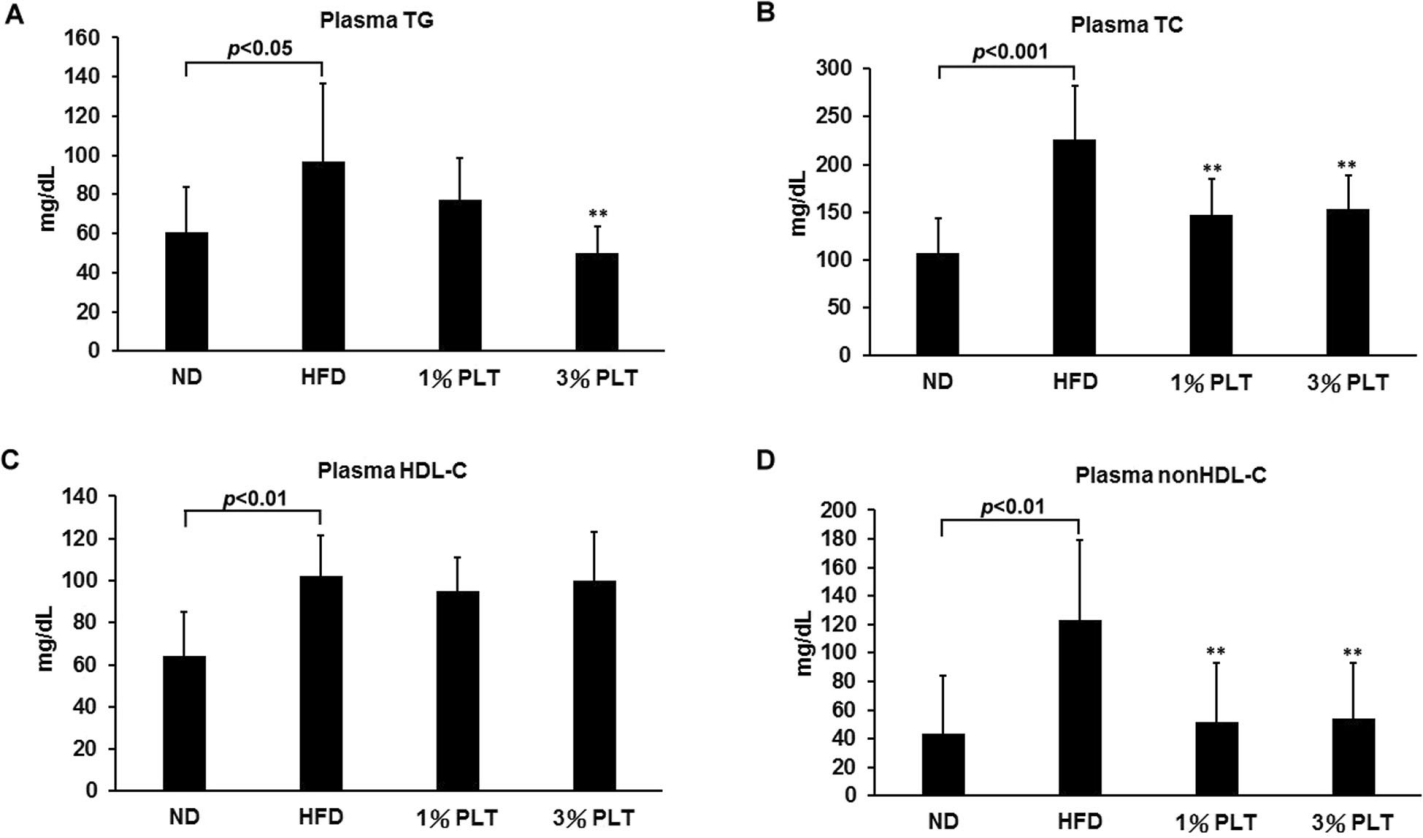
The effect of PLT on plasma lipid levels in C57BL/6 J mice fed an HFD. The levels of **a** plasma triglyceride (TG), **b** total cholesterol (TC), **c** high density lipoprotein (HDL-C) and **d** non-high density lipoprotein (non-HDL-C). Data are shown as means ± SEM. * *p* < 0.05; ** *p* < 0.01; *** *p* < 0.001 vs. HFD

To examine the effect of PLT on the lipid composition of the serum, the plasma TG, TC, HDL-C, and non-HDL-C levels were monitored. The TG, TC, HDL-C and non-HDL-C levels were significantly higher in the HFD group than the ND group (Fig. 4a, b, c, and d). In the HFD-3% PLT group, plasma TG level was significantly lower (Fig. 4a). Plasma TC and non-HDL-C were significantly lower in the HFD-PLT groups, except for plasma HDL-C (Fig. 4b, c, and d). This suggests the presence of hypertriglyceridemia and high cholesterol phenomena in the HFD mouse model, which is consistent with the symptoms of obesity in humans. The findings also indicate the inhibition of hyperlipemia bioactivity by PLT.

### The incidence of hepatic steatosis under high fat conditions was prevented by PLT treatment

**Fig. 5:**
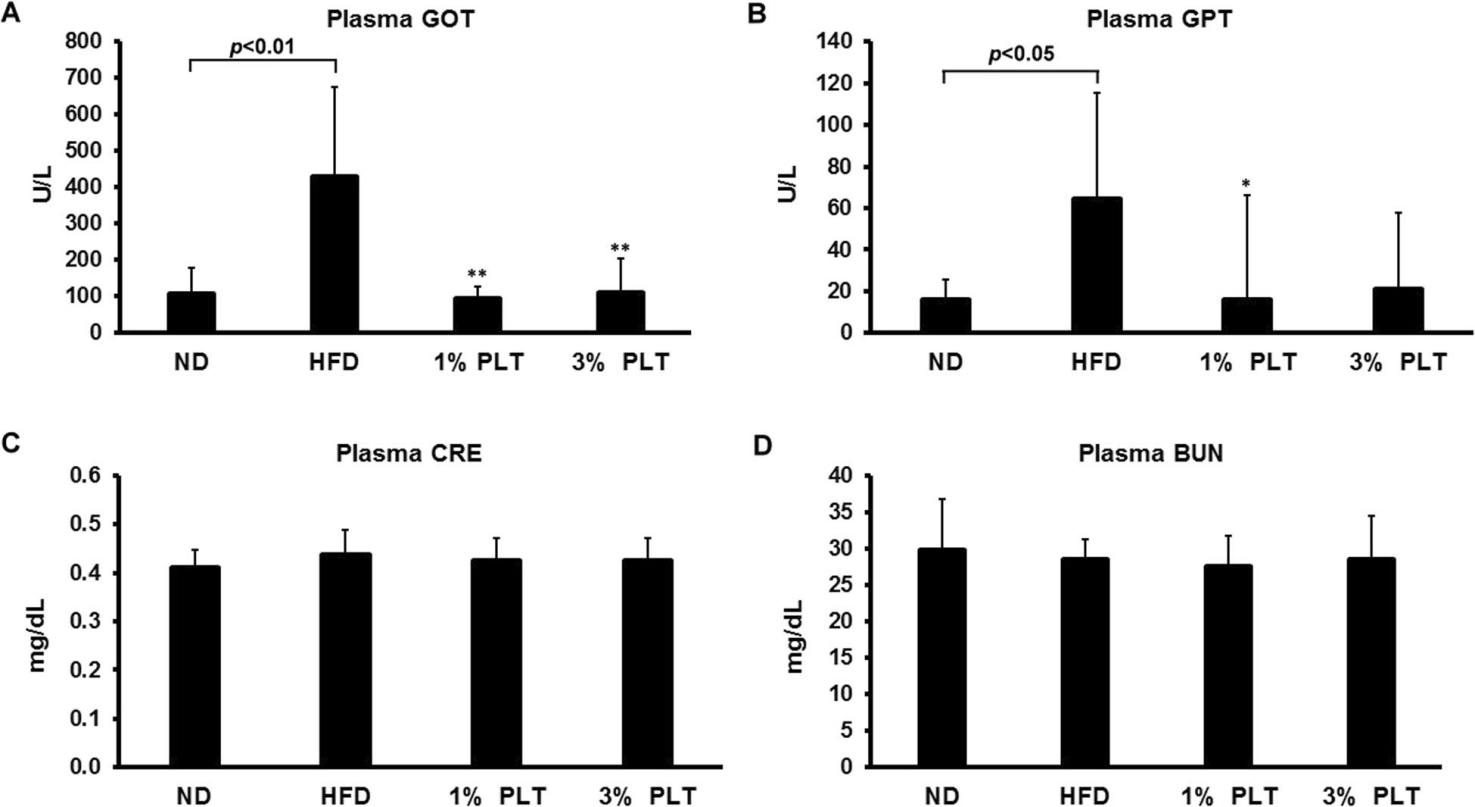
The effect of PLT extract treatment on the level of hepatic steatosis-related marker in C57BL/6 J mice fed an HFD. The plasma levels of the hepatic lipotoxicity markers **a** aspartate aminotransferase, AST/GOT, **b** alanine aminotransferase, ALT/GPT. The plasma levels of the kidney lipotoxicity markers **c** creatinine, CRE, and **d** blood urea nitrogen, BUN. **e** The plasma levels of the pancreas lipotoxicity marker, lipase (LIP). Data are shown as means ± SEM. * *p* < 0.05; ** *p* < 0.01; *** *p* < 0.001 vs. HFD

It was shown previously that a high-fat diet promotes hepatic steatosis and affects the β-oxidation status and balance of oxidants, which has effects on body weight, insulin signaling and other metabolic parameters [22]. Therefore, we measured several hepatic-steatosis markers to evaluate the incidence of steatohepatitis. Levels of aspartate aminotransferase (AST/GOT) and alanine aminotransferase (ALT/GPT) in serum were measured as markers of hepatic lipotoxicity and indication of hepatic tissue injury [23, 24]. We found the levels of plasma GOT and GPT were increased in HFD mice but this increase was prevented by PLT treatment (Fig. 5a and b). However, renal disease creatinine (CRE) and blood urea nitrogen (BUN) did not differ significantly between the groups (Fig. 5c and d). Similarly, the plasma levels of the pancreas lipotoxicity marker, lipase (LIP), did not vary significantly among the groups (Fig. 5e). This suggests that PLT prevented diet-induced hepatic steatosis.

### PLT consumption prevents obesity-driven dysbiosis of the gut microbiota in HFD mice

**Fig. 6:**
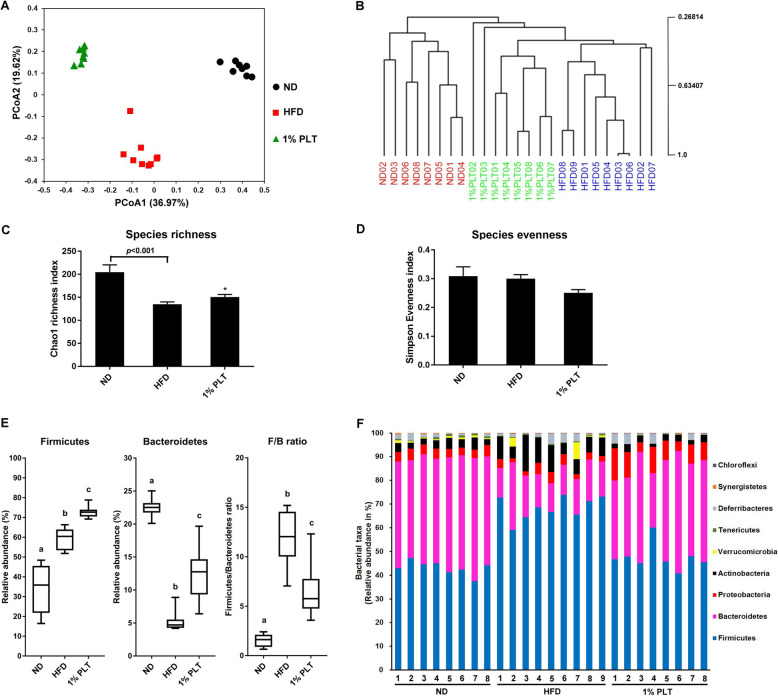
PLT modulated the composition of the HFD-disrupted gut microbiota. **a** Principle coordinate analysis (PCoA) of gut microbiota based on OTU abundance, **b** multivariate analysis of variance from the PCoA matrix score, **c** bacterial richness (Chao1 Richness Index), **d** bacterial evenness (deduced from the Simpson Index), **e** the relative abundances of Firmicutes, Bacteroidetes, and the ratio of Firmicutes to Bacteroidetes, **f** bacterial taxonomic profiling at the phylum level of gut microbiota. Diversity indexes are expressed as the mean from subsampled datasets ± SEM. Data with different superscript letters are significantly different at *p <* 0.05 according to the one-way analysis of variance statistical analysis

**Fig. 7:**
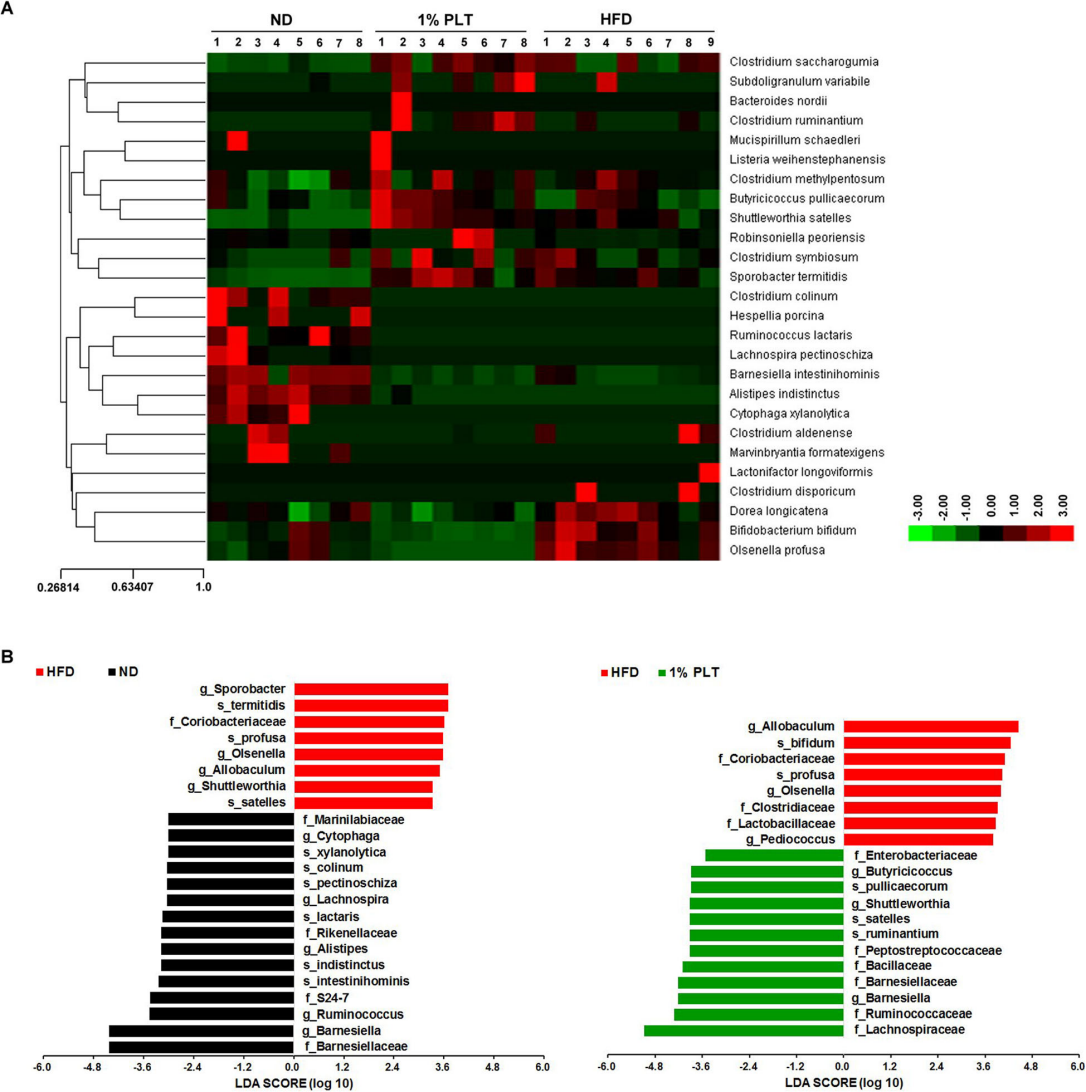
PLT changes in gut microbial populations in HFD mice. **a** Heatmap showing the abundance of 26 OTUs significantly altered by PLT in the HFD-fed mice, based on RDA. **b** Linear discrimination analysis (LDA) effect size (LEfSe) was calculated to explore the taxa within the lowest taxonomic level possible that more strongly discriminate between the gut microbiota of ND vs. HFD and 1% PLT vs. HFD

Operational taxonomic unit (OTU)-based principle coordinates analysis (PCoA) demonstrated distinct clustering of the microbiota compositions of the different groups (Fig. 6a). Multivariate analysis of variance of PCoA also revealed significant separation between the ND, HFD-PLT, and HFD groups (Fig. 6b). Although there is no statistical difference of microbial species evenness between the different groups, the species richness of the gut microbiota in the HFD mice was significantly less than that in the ND mice (Fig. 6c and d). Administration of PLT increased the microbial species richness of the mice fed with an HFD (Fig. 6c). The microbial composition of the faeces was significantly altered by an HFD, with Firmicutes relative abundance increased and Bacteroidetes relative abundance decreased in the HFD group (Fig. 6e). The rise of the Firmicutes to Bacteroidetes ratio caused by an HFD was attenuated by administration of PLT. Taxonomic profiling showed the composition of the microbiota varied among the different groups and was dominated by Firmicutes and Bacteroidetes at the phylum level (Fig. 6f). Because the Firmicutes and Bacteroidetes are the two hallmarks of obesity-driven dysbiosis, the findings implied that PLT prevented microbial dysbiosis and impeded the reduction of microbial richness in the HFD mice. A heat map of the scores of the relative abundances of the different groups, based on RDA, is shown in Fig. 7a. The relative abundances of 26 OTUs that were altered by PLT, including the heatmap of the relative abundance (log10 transformed), and the changing direction of represented bacterial taxa information (species level) modulated by PLT are shown. We then calculated linear discriminant analysis (LDA) scores for the most discriminating OTUs of the different groups. The LDA effect size (LEfSe) was computed to explore the taxa within the lowest taxonomic level possible. We found that the mean abundance of 25 OTUs differed significantly between the HFD and ND groups, and a total of 16 OTUs was more abundant in the ND group (Fig. 7b left). Among the most prominent, f_Barnesiellaceae, g_Barnesiella, f_Ruminococcaceae, and f_Lachnospiraceae were discriminating faecal bacterial communities of the PLT mice that differed from the HFD mice (Fig. 7b right). PLT treatment was effective at modulating the gut microbiota induced by HFD.

## Discussion

Dietary food has been shown to have potential effects in terms of regulating blood glucose, blood lipid and weight, which are parameters for evaluating metabolic syndrome. Purple-leaf Tea (*Camellia sinensis* L., PLT) was once popular as a beverage and food supplement in China and Vietnam. Flavonoids and their subgroup, anthocyanins, are responsible for the pigments of red, blue, and purple in fruit and vegetables, such as blueberries, grapes, and red onions, in which their concentrations are elevated and mask the green color of chlorophylls. Previous studies reported that the content of anthocyanins in purple leaves is more than 400 times greater than in green leaves [25]. It also was shown that anthocyanin-rich PLT has antioxidant and antimicrobial effects and may be a potential dietary compound to prevent lifestyle related disorders and certain cancers [7, 8, 26].

The development of metabolic disorders, such as diabetes and fatty liver diseases, has been shown to be closely correlated with visceral obesity and diet [19, 22]. In fact, tea and tea extracts have been used as supplements for weight management and control of metabolic complications for many years [3–6]. In this study, we established a mouse model of metabolic syndromes by feeding mice with a high fat diet (HFD) and tested the effects of purple-leaf tea on these mice. We found that HFD mice showed greater food efficiency ratios (FERs) than normal diet (ND) mice and administration of PLT protected the HFD mice significantly against increased FERs. HFD mice also exhibited increased adipose tissue mass, with adipocytes of greater diameter, and supplementation with PLT was able to prevent lipid deposition in these mice. These findings infer that PLT may play a critical role in preventing visceral obesity. We found HFD mice had significantly higher fasting blood glucose levels and greater glucose tolerance than ND mice, suggesting an abnormality of insulin function in HFD mice. PLT was able to maintain fasting blood glucose and glucose tolerance at normal levels in the HFD mice. Previous studies showed that increased intake of anthocyanins and flavone is associated with lower insulin resistance [27]. We suspected that the protective effect against metabolic syndrome in our model may be due to the abundance of flavonoids and anthocyanins in PLT to counter systematic hyperlipedema and hyperglycemia. PLT may ameliorate metabolic syndrome and improve insulin sensitivity caused by diet.

Anthocyanins have been shown to have a role in the modulation of lipid homeostasis in various organs, including the liver [9]. Because the liver is a crucial metabolic organ and governs gluconeogenesis and lipogenesis, we investigated the effects of PLT on fatty liver and lipemia syndrome in our HFD mouse model. We found that high-fat diet induced lipid accumulation in the liver and upregulated the hepatic triglyceride (TG), plasma TG, hepatic cholesterol, and plasma cholesterol levels of the mice. Levels of plasma GOT and GPT also were upregulated in the HFD mice. These markers of hepatic steatosis were maintained at normal levels with PLT treatment, which implies PLT protected the HFD mice from diet-induced hepatic steatosis. A previous study using fatty-acid-treated HepG2 cells demonstrated that anthocyanins reduce lipid accumulation by inhibition of lipogenesis or promotion of lipolysis [28]. Our findings suggest that anthocyanins-rich PLT was able to inhibit fatty liver and hyperlipemia bioactivity by promoting lipolysis.

Kerio et al. studied the free radical-scavenging activity of the tea extracts and found anthocyanins played an important role in the antioxidant activities in vitro [25]. Previous studies have shown the amounts and subtypes of anthocyanin in PLT [25, 29, 30], as well as the gene analysis by He et al. [31] and metabolic analysis by Shen et al. [32]. Four of the major anthocyanins in PLT are cyaniding-, cyaniding-glucoside, delphinidine-, and delphinidine acylated with glucose. However, more subtypes of flavonoids in PLT providing significant effect of PLT on diet-induced metabolic syndrome cannot be excluded. Thus, the active constituents in PLT need further identification and quantification.

The GI tract is the first organ that is exposed to the diet. Recent studies of obesity-related disorders in HFD mice suggest that the functionality, integrity and microbial complexity of the GI tract contribute to local and systemic health [11, 12]. We inferred that the beneficial effects of PLT may be attributed to the metabolism of flavonoids or anthocyanins by gut microbiota, and lead to an alteration of microbial composition. In our study, pyrosequencing-based analysis of bacterial 16S rRNA in the faeces of the mice revealed that PLT treatment altered the gut microbiota of the HFD mice, maintaining the diversity of the gut microbiota. Interestingly, various studies have shown that lean individuals have a greater microbial diversity than obese individuals [11, 12]. The results of our study suggest that PLT might modulate lipid accumulation and weight-loss by maintaining the diversity of gut microbiota.

Diet and the use of antibiotics and probiotics affect the composition of intestinal microbiota and the balance of microbiota is critical to the integrity of the colonic barrier [33, 34]. The intestinal barrier protects the mucosal tissues against pro-inflammatory molecules, microorganisms and antigens. Microbial dysbiosis and damage to the integrity of the colonic barrier may induce translocation of bacterial products, such as lipopolysaccharide (LPS), into the blood, which may result in activation of Toll-like receptor 4 (TLR4) and lead to inflammatory responses, insulin resistance, and obesity [35]. It has been reported that an HFD could increase intestinal permeability, possibly through modification of the microbial population [36]. The leaky intestines of HFD mice may result in the LPS of Gram-negative bacteria entering the enterohepatic circulation, causing inflammation. Studies have shown that chronic inflammation is associated with obesity, and obese individuals appear to have increased levels of Gram-positive Firmicutes over Gram-negative Bacteroidetes [37, 38]. We observed that the ratio of Firmicutes to Bacteroidetes (F:B) ratio was higher in HFD mice, and supplementation with PLT was able to maintain a normal ratio F:B. This observation suggests the anti-obesity effects of PLT may be attributable to maintenance of a normal bacterial population.

In our study, linear discriminant analysis (LDA) scores revealed that the bacterial community of PLT and HFD mice was similar. Interestingly, the relative abundance of the family *Ruminococcaceae* was higher in the PLT and ND groups than the HFD group. A previous study showed that the diet shifts the diversity of dominant gut bacteria and the population of Gram-positive *Ruminococcaceae* declines in mice fed with high-fat diet [36]. It was also shown that obese patients with non-alcoholic steatohepatitis have a lower abundance of the family *Ruminococcaceae* than healthy individuals [39, 40]. These previous results are in agreement with our findings that HFD mice had symptoms of steatohepatitis and a low abundance of *Ruminococcaceae. Ruminococcaceae* are commonly found in the mammalian gut and can degrade cellulose and hemicellulose from plants. The degraded compounds then are fermented and converted into short chain fatty acids (SCFAs). SCFAs have been shown to have the ability to protect against inflammation [41]. These findings suggest that the anti-inflammatory effects of phenolic/anthocyanin involve the interaction between local microbiota and digested compounds. Therefore, prevention of microbial dysbiosis and obesity by PLT might be through an increase in the populations of beneficial species, such as *Ruminococcaceae.*

## Conclusions

To sum up, in our mouse model, PLT contributed to the maintenance of a normal body weight after a 10-week high fat diet. In addition, food intake efficiency was kept low and fat accumulation that may cause metabolic syndrome was prevented. This effect on metabolism was examined through the concentrations of glucose, lipids, and other insulin resistance-associated factors. Administration of PLT modified the host microbial composition and intestinal integrity, maintaining gut homeostasis. These solid results indicate that PLT has promising bioactivity in regulating obesity, insulin sensitivity, hyperlipidemia and hepatic steatosis by maintaining the composition of the gut microbiota.

## Data Availability

The datasets used and/or analyzed during the current study available from the corresponding author on reasonable request.

## Abbreviations

BUN: Blood urea nitrogen
CRE: Creatinine
EAT: Epididymis adipose tissue
FER: Food efficiency ratios
GOT: Glutamic oxaloacetic transaminase
GPT: Glutamic pyruvic transaminase
HDL-C: HDL-cholesterol
HFD: High fat diet
IPGTT: Intraperitoneal glucose tolerance test
LIP: Lipase
OTU: Operational taxonomic unit
ND: Normal diet
PLT: Purple-leaf Tea
TG: Triglyceride
TC: Total cholesterol
